# Post-marketing safety surveillance and re-evaluation of Xueshuantong injection

**DOI:** 10.1186/s12906-018-2329-z

**Published:** 2018-10-16

**Authors:** Chunxiao Li, Tao Xu, Peng Zhou, Junhua Zhang, Ge Guan, Hui Zhang, Xiao Ling, Weixia Li, Fei Meng, Guanping Liu, Linyan Lv, Jun Yuan, Xuelin Li, Mingjun Zhu

**Affiliations:** 1 The First Affiliated Hospital of Henan University of Chinese Medicine, Zhengzhou, 450000 People’s Republic of China; 2 Evidence-based Medicine Center, Tianjin University of Traditional Chinese Medicine, Tianjin, 300000 People’s Republic of China; 3 Guangxi Wuzhou Pharmaceutical (group) Co., Ltd, Wuzhou, 543000 People’s Republic of China; 4 Shanghai Yongzheng Medical Science and Technology Co., Ltd, Shanghai, 200000 People’s Republic of China

**Keywords:** Xueshuantong injection, Post-marketing, ADRs/ADEs, Traditional Chinese medicine injections

## Abstract

**Background:**

Traditional Chinese medicine injections (TCMIs) have been widely used to treat severe and acute diseases due to their high bioavailability, accurate curative effect, and rapid effect. However, incidence rates of adverse drug reactions (ADRs) of TCMIs have also increased in recent years. Xueshuantong injection (XSTI) is a commonly-used TCMI comprised of Panax notoginseng total sapiens for the treatment of stroke hemiplegia, chest pain, and central retinal vein occlusion. Its safety remains uncelar. Therefore, post-marketing safety of XSTI was studied in this research.

**Methods:**

In present study, post-marketing safety surveillance and re-evaluation of XSTI were reported. Thirty thousand eight hundred eighty-four patients in 33 hospitals from 7 provinces participated in this study. Incidence rate, most common clinical manifestations, types, severity, occurrence time, and disposal of ADRs were calculated.

**Results:**

Incidence rate of ADR of XSTI was 4.14‰ and the most common clinical manifestations were skin and its appendages damage. Type A accounts for 95.49% of ADRs of XSTI and most of them (41.41%) were occurred within 24 h after receiving XSTI treatment. Severities of most ADRs of XSTI were moderate reactions (86.72%). Main disposition of ADRs of XSTI was drug withdrawal and symptomatic treatment (54.69%).

**Conclusions:**

Our data provide basis for improvement of instructions of XSTI and clinical safety of XSTI. Post-marketing surveillance of TCMIs in this study is a powerful tool to identify types and manifestations of ADRs to improve safety and effectiveness of drugs in clinical applications.

**Trial registration:**

This protocol has international registration in China clinical trial registration center (ChiCTR~OPC~ 14,005,718) at December 22, 2014.

## Background

Traditional Chinese medicine injection (TCMI) is made by modern technologies and scientific methods to extract and purify effective substances from herbs (or decoction pieces). Compared with other traditional Chinese medicine formulations, injection has advantages of high bioavailability, rapid, and accurate curative effect. Therefore, TCMI is widely used to treat many severe and acute diseases [1–7]. In recent years, with the widespread use of TCMIs, the incidences of adverse drug reactions (ADRs)/adverse drug events (ADEs) has gradually increased [8–10]. However, safety profile of most TCMIs remains largely unknown currently.

Xueshuantong injection (lyophilized) (XSTI) is a standardized herbal preparation and has been collected by “2012 national essential drugs list” and People’s Republic of China Pharmacopoeia, respectively. Notoginseng total saponins, isolated from the root and rhizome of *P. notoginseng*, is the main component of XSTI. XSTI is generally used for treatment of cardiovascular and cerebrovascular disease [11]. Total revenue of XSTI in Chinese market in 2013 was over $700 million [12]. Therefore, the enormous consumption requires stricter and accurate evidence on its safety. However, many reports on the ADRs of XSTI were case reports and there is still lack large sample and high level evidence-based basis for safety of XSTI. Till now, evaluation on post-marketing safety of XSTI has not been reported. Therefore, ADRs/ADEs of XSTI were studied in this research using hospital centralized monitoring method.

Hospital centralized monitoring also known as real world study (RWS), is an observational research method by recording detailed ADRs of drugs within a certain range of a hospital or an area in a certain period of time. It is attracting more and more attention in field of global clinical epidemiology due to its broad range of inclusion and exclusion criteria, comprehensive coverage of population, and authenticity [13–15]. A new hospital centralized monitoring method based on hospital information system (HIS) system was established in. our previous study on post-market clinical safety evaluation of TCMI [16]. In present research, post marketing safety (including incidence rate, types, severities, and other information of ADRs/ADEs) of XSTI with 30,884 cases by employing an improved method of hospital-centralized monitoring. This research is the first post-marketing ADRs/ADEs study of XSTI with large scale and multi-center and can provide essential basis for safe clinical use of XSTI.

## Methods

### Inclusion and exclusion criteria

Inclusion criteria: patients who used XSTI.

Exclusion criteria: patients who did not use XSTI.

### Subjects

A total of 30,884 in-patients received XSTI from 33 hospitals in 7 provinces participated in this study between January 1, 2015 and December 31, 2016.

### Drug

All three product specification (100 mg、150 mg and 250 mg per bottle) of XSTI were manufactured by Guangxi Wuzhou Pharmaceutical Co., Ltd. (Wuzhou, Guangxi, China). All drugs used in this research were sold on the market and in conformity with the standard of Ministry of Public Health of China.

### Method design

This study was not a randomized controlled trial but a centralized monitoring study in hospital and all data were collected from clinical daily treatment without any intervention. Thus this study was not designed entirely according to CONSORT guidelines. We designed the monitoring data collection and quality control method according to other hospital centralized monitoring methods [13–16].

#### Method of monitoring data collection

**Fig. 1:**
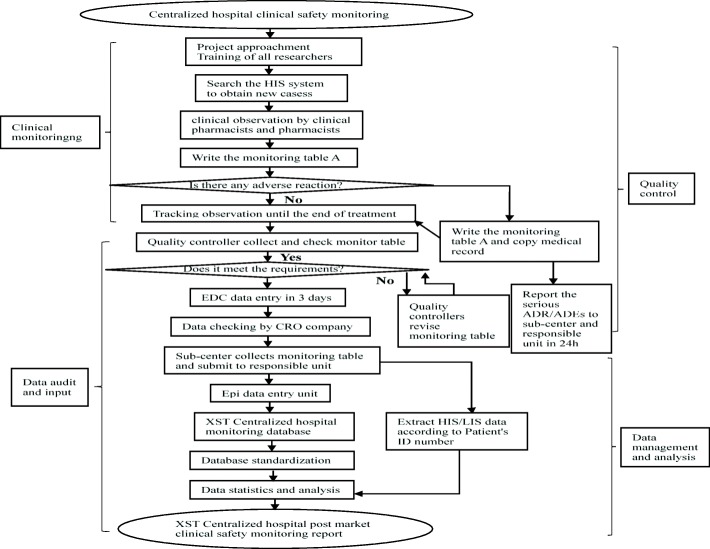
Post-marketing safety re-evaluation of XSTI workflow

**Fig. 2:**
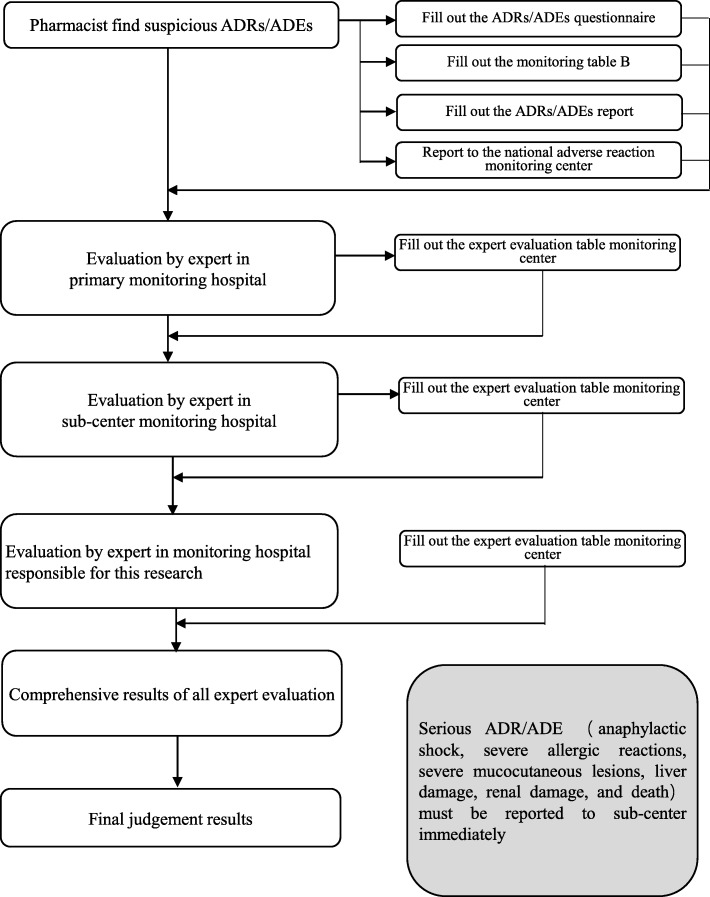
Treatment flowsheet of ADRs/ADEs

The monitoring data were from two parts: monitoring table and hospital information system / laboratory information management system (HIS/LIS). Information in front page of the medical record, doctor’s orders and results of laboratory examination were extracted from HIS/LIS system after being approved by ethics committee. To ensure the safety of the patient’s personal information all monitors have been trained on information confidentiality. Monitoring table consists of Table A (basic monitoring information including daily dose, frequency, drug combination, etc.) and Table B (ADR/ADE information). Table A was filled by pharmacists within 5 days after the end of medication by “face-to-face” observation. Monitoring Table B was filled once ADR/ADE, especially serious ADR/ADE such as anaphylactic shock, severe allergic reactions, severe mucocutaneous lesions, liver damage, renal damage, and death, was happened. Accordance to requirements of “National ADR Reporting and Monitoring Management Measures”, all serious ADRs/ADEs were further investigated by a panel consisting of head of organizer of the project and staffs from sub center and manufacturing enterprise. “Adverse Drug Reaction / Event Report” was written and submitted to official website according to the rules of the CFDA. The overall data collection flow chart is shown in Fig. 1 and ADRs/ADEs processing process is shown in Fig. 2.

#### Method of monitoring quality control

**Fig. 3:**
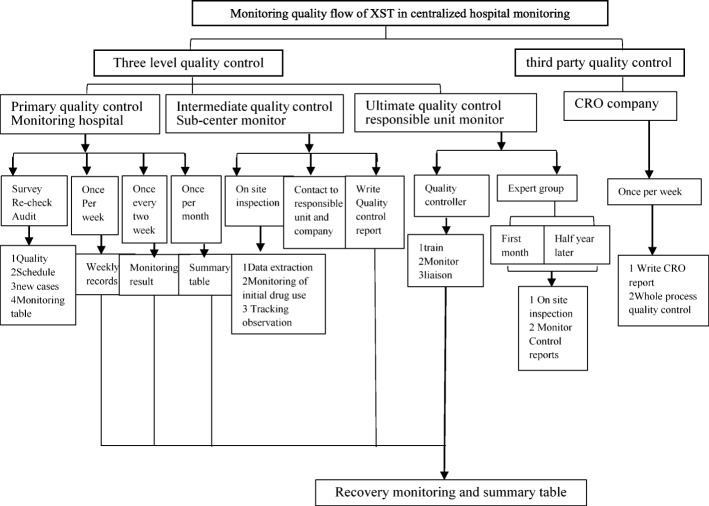
Monitoring quality control workflow

In order to guarantee the objectivity and accuracy of ADR results, unified training on monitoring plan and ADRs/ADEs judgment was carried out for monitoring personnel and a three-grade evaluation of ADRs / ADEs and third party quality control were conducted in this study. The detailed monitoring process is shown in Fig. 2. Strict selection criteria were set for the screening of participating hospital. Primary quality control monitoring hospital included comprehensive hospital and traditional Chinese medicine hospital. Sub-center monitoring hospitals were all three grade hospital in China and have organized or participated in the evaluation of drug safety. All participating hospitals had a team of clinical pharmacists and collected at least 500 cases within 1 years. There were 7 sub centers in total, and each sub center was responsible for 5–6 hospitals. A contract research organization (CRO) company (Shanghai Yongzheng medical science and Technology Co., Ltd.), was employed to carry on quality management of the study (Fig. 3). Reliability of monitoring reports and research progress of each monitoring hospitals and monitoring centers regularly were judged by CRO company. The hospitals which couldn’t complete the monitoring progress on time or their monitoring reports were judged as unqualified more than three times were refused to continue to participate into the research project. The sub center was eliminated when more than half of its monitoring hospitals were eliminated.

### Correlation assessment between ADRs and ADEs

Correlation assessment between ADRs and ADEs was conducted according to method recommended by CFDA evaluation center of adverse reactions. All ADRs/ADEs were preliminarily classified on basis of their definitions, respectively. ADR is unrelated or unexpected adverse reaction to medication purpose when using approved drugs within normal dosage. It does not include reactions caused by accidental or intentional drug overdoses or improper medications. ADE refers to any injury occurred during drug administration period, whether or not drug usage is the cause of injury. ADR is a special type of ADE for which the causative relationship between drug usage and adverse reaction is identified. Relevance assessment is divided into 6 grades: (1) Certain: the sequence between medication and ADRs’ occurrence is reasonable. ADRs could be stopped or quickly reduced or turn better after drug withdrawal. Alternatively, ADRs would be occured again or significantly worse when drug was re-administered. It could also be supported by literatures. Notably, primary disease and other factors should be ruled out. (2) Probable: there is no history of repeating medication, others are same as “Certain”. If the investigated drug was administrated in combination with other drugs, the probability of ADR caused by combined drugs could be excluded. (3) Possible: there is close relationship between medication and ADEs’ occurrence. It is coincided with common type of ADRs, but there is no reaction data after drug withdrawal, or there are more than one drug leading to ADRs/ADEs, or causative factors of primary disease could not be ruled out. (4) Unlikely: there was no close relationship between medication and ADEs’ occurrence. The reactions do not link to ADRs/ADEs of the investigated drug. Reactions during development of primary disease may display similar clinical manifestations. (5) Pending: There are missing contents of “Monitoring Information Form” and evaluation will not be completed until the supplementary specifications are provided. Thus, it is difficult to determine relationship between cause and effect due to absence in documentation. (6) Unassessable: many items in the “Monitoring Information Form” are unavailable. It is unable to analyze relationship between cause and effect because missing items could not be supplemented [2, 17, 18].

## Results

### Number of cases of XSTI in each hospital

In this study, a total of 30,884 cases received XSTI from 33 hospitals participated in the monitoring assessment. The number of ADE cases in 33 monitoring hospitals is shown in Table 1.

**Table 1:** The number of ADE cases in 33 monitoring hospitals

Hospitals	Number of cases	Constituent ratio (%)
The First Affiliated Hospital of Henan University of TCM	2103	6.81
The Second Affiliated Hospital of Henan University of TCM	1006	3.26
Henan Province People’s Hospital	1997	6.47
Luohe Central Hospital	697	2.26
General Hospital of Shenma medical group	1089	3.53
The First Affiliated Hospital of Guangxi University of TCM	1500	4.86
The First people’s Hospital of Nanning	1011	3.27
The people’s Hospital Guangxi Zhuang Autonomous Region	601	1.95
Nanning Hospital of TCM	607	1.97
The Jiangbin Hospital Guangxi Zhuang Autonomous Region	600	1.94
Wuzhou Red Cross Hospital	812	2.63
Wuzhou People’s Hospital	802	2.60
The Second Affiliated Hospital of Tianjin University of TCM	1810	5.86
Peking University Binhai Hospital	515	1.67
Tianjin Huanhu Hospital	1203	3.89
Tianjin First Central Hospital	1213	3.93
Tianjin Hospital of ITCWM Nankai Hospital	802	2.60
Shuguang Hospital of Shanghai University of TCM	501	1.62
Tongji Hospital of Shanghai	501	1.62
Longhua Hospital of Shanghai University of TCM	502	1.63
People’s Hospital of Pudong District of Shanghai	504	1.63
West China Hospital of Scichuan University	1504	4.87
Sichuan Cancer Hospital	1001	3.24
The people’s Hospital of Dujiangyan	800	2.59
Affiliated Hospital of Chengdu University	603	1.95
Affiliated Hospital of Chengdu University of TCM	1034	3.35
Gansu Province Hospital of TCM	1223	3.96
The Second People’s Hospital of Gansu	406	1.31
The People’s Hospital of Gansu	807	2.61
Affiliated Hospital of Gansu University of TCM	635	2.06
The First Bethune Hospital of Jilin University	1830	5.93
Affiliated Hospital of Changchun University of TCM	502	1.63
The General Hospital of CNPC in Jilin	163	0.53
Total	30,884	100.00

### Association assessment of adverse reactions

In this study, 128 cases were grouped as “probable and possible “. Results of relevance evaluation were shown in Table 2.

**Table 2:** Results of correlation evaluation between ADRs and ADEs of XSTI

Results of correlation evaluation	Number of cases	Constituent ratio (%)
Certain	0	0.00
Probable	53	41.09
Possible	75	58.14
Unlikely	1	0.78
Pending	0	0.00
Unassessable	0	0.00
Total	129	100

### Incidence rate and manifestations of ADRs

The ADR incidence of XSTI was 4.14‰. The clinical manifestations were 236 times. The most common clinical manifestations were skin and its appendages damage (52.97%), systemic injury (9.32%), and central and peripheral nervous system damage (8.90%). Statistics analysis were shown in Table 3 in detail.

**Table 3:** ADR manifestations of XSTI

Systems/organs	Number of cases/incidence rate ‰	Constituent ratio (%)	Manifestations (number of cases/frequency‰)
Skin and its appendages	125/4.05	52.97	erythra (61/1.98), pruritus (50/1.62), maculopapule (8/0.26), hyperhidrosis (4/0.13), urticaria (4/0.13)
Systemic injury	22/0.71	9.32	fever (6/0.19), Shiver (6/0.19), edema (4/0.13), Chest pain (2/0.06), anaphylactoid reaction (1/0.03), periorbital edema (1/0.03), hot flush (1/0.03), acratia (1/0.03)
Central and peripheral nervous system damage	21/0.68	8.90	headache (9/0.29), giddy (6/0.19), paresthesia (4/0.13), lower limb spasticity (1/0.03), tremor (1/0.03)
Gastrointestinal system damage	14/0.45	5.93	sicchasia (4/0.13), vomit (2/0.06), hemorrhage of gastrointestinal tract (1/0.03), aggravation of gastrointestinal bleeding (1/0.03), dry lips (1/0.03), Stool discoloration (1/0.03), abdominal pain (1/0.03), discoloration of tongue (1/0.03), flatulence (1/0.03), toothache (1/0.03)
Respiratory system damage	14/0.45	5.93	Chest tightness (10), dyspnea (2), laryngeal spasm (1/0.03), epistaxis (1/0.03)
Extra cardiac vessel damage	10/0.32	4.24	flushing (9/0.29), phlebitis (1/0.03)
Medication site damage	10/0.32	4.24	local numbness (4/0.13), injection site pain (4/0.13), injection site numbness (1/0.03), injection site pruritus (1/0.03)
Urinary system damage	7/0.23	2.97	facial edema (6/0.19), hematuria (1/0.03)
Heart rate and arrhythmia	6/0.19	2.54	palpitation (5/0.16), tachycardia (1/0.03)
Nerve disorders	4/0.13	1.69	feel suffocated (3/0.10), insomnia (1/0.03)
Visual impairment	1/0.03	0.42	abnormal tears (1/0.03)
General cardiovascular system damage	1/0.03	0.42	Hypertension (1/0.03)
latelets and bleeding, coagulopathy	1/0.03	0.42	Gingival bleeding (1/0.03)
Total	236	100.00	

Incidence rate = number of adverse events *1000‰/ total number of cases

### Types of ADRs

ADRs are classified into three types (types A, B, and C) by WHO. Type A reaction caused by the enhancement of pharmacological effect of drugs is dose-related and able to be predicted. Type B reaction is abnormal reaction unrelated to normal pharmacological effect. It is not able to be detected by conventional toxicological screening and hard to make prediction. Type C reaction refers to abnormal reaction other than types A and B. According to above classification, all 128 cases of ADRs were divided into type A, B, and C, respectively as shown in Table 4.

**Table 4:** Comprehensive evaluation of occurrence types of ADR/ADE

Type	Number of cases	Constituent ratio (%)
A	127	95.49
B	6	4.51
C	0	0.00
Total	133	100

### Time of occurrence of ADRs

Time of occurrence of ADRs after injection of XSTI is shown in Table 5 and Table 6. According to the results, most ADRs of XST were occurred rapidly and nearly half cases of ADRs were appeared on the first day of injection (41.41%). There is 25.78% of ADRs were happened 2~ 4 days after injection.

**Table 5:** ADR Occurrence time of XSTI

	occurrence time (hours)				Total
	Day 1	Day 2~ 4	Day 5~ 7	>7 Days	
Number of cases	53	33	18	24	128
Constituent ratio (%)	41.41	25.78	14.06	18.72	100.00

**Table 6:** The time distance of ADR occurrence time and the last medication time of XSTI

	occurrence time (hours)				Total
	<0.5	0.5~ 1	1~ 12	12~ 24	
Number of cases	33	23	53	19	128
Constituent ratio (%)	25.78	17.97	41.41	14.84	100.00

### Severity of ADRs

As shown in Table 7, severity of ADRs were classified into three grades: mild (symptoms or signs can be felt and stopping medication or special treatment is no necessary), moderate (symptoms and signs are tolerable and there is no effect on daily life but special treatment is necessary), and severe (symptoms and signs are intolerable and drug withdrawal and special treatment are needed). Our results showed that most cases (86.72%) were graded into moderate reactions and 11 cases were classified as mild. In addition, among 6 cases with severe ADRs, 3 cases had severe symptoms, including rash, flushing, shivering, palpitation, high fever, dyspnea, and convulsions.

**Table 7:** The severity classification of ADR of XSTI

Severity classification	Number of cases	Constituent ratio (%)
Mild	11	8.59
Moderate	111	86.72
Severe	6	4.69
Total	128	100.00

### Disposal of ADRs

In most cases, ADRs need special treatments such as reducing times and dose of usage, withdrawal, symptomatic treatment, or combined together. Special treatments used in this study were listed as follows: withdrawal and symptomatic treatment (54.69%), withdrawal (30.47%), symptomatic treatment (7.03%) (Table 8). In addition, 1.56% of the ADRs did not get any special treatment.

**Table 8:** The disposal of ADR of XSTI

Disposal	Number of cases	Constituent ratio (%)
None	2	1.56
Reduce dripping speed (RDS)	7	5.47
Reduce dose	0	0.00
Drug withdrawal (DW)	39	30.47
Symptomatic treatment (ST)	9	7.03
RDS + DW + ST	1	0.78
DW + ST	70	54.69
Total	128	100.00

### Recovery of ADRs

Among of patients with ADRs, 80 cases were cured, 48 cases got improved, and there was no sequelae and death. Upon recovery time, 12, 26, 17, and 70 cases were improved within 1 h (9.38%), 1 ∼ 6 h (20.31%), 6 ∼ 24 h (13.28%), or over 24 h (54.69%), respectively. In addition, 3 cases were not recorded in detail.

## Discussion

The incidence of ADRs of XSTI was 4.14‰, which was an “occasionally” “level and severe ADRs was 0.19 ‰, which was a “rare” grade and 95.49% of ADRs in this research were type A which can be predicted. 42.97% of ADRs were recovered in 24 h and there was no sequelae and death. Therefore, XSTI is safe in clinical use according to the incidence, type and recovery of ADRs in this study. Most of ADRs were anaphylaxis which indicated that safety monitoring should be in progress promptly. In addition, ADRs of XSTI could happened throughout the whole course of treatment after administration which indicated that the whole process needs safety monitoring. So far, the description of the XSTI used in the study is not clear about ADRs. The results of this study make clear the manifestations of ADRs of XSTI, which provide a high level evidence-based basis for the improvement of the instructions. In this research, manifestations of ADRs were studied and the most common ADR manifestation of XSTI was skin and its appendages damages which were consistent with other published reports [19]. However, there is no suitable medicine to alleviate the ADRs of cutaneous systems. The main preventive measures by far is washing infusion tube before the injection of different injections to reduce the incidence of ADRs during the combined use of XSTI and other injections. It is generally believed that the skin and its appendages damages were caused by allergies. There is a study using P815 cell degranulation model to screen components of XSTJ [20]. Results showed that XSTI promoted the P815 cell degranulation and the effect may have related to Ginsenoside Rb1 and Rg1. Besides, impurities are difficult to remove in purification and refining processes due to the complex components in traditional Chinese medicine injections, which may also cause anaphylaxis [21–23]. However, few studies on the anaphylaxis mechanism of XSTI or panax notoginseng saponins and further investigation are needed to explore the ADR mechanism of XSTI. In addition, this study found some ADRs beyond instruction.

By comparison of four methods (hospital-centralized monitoring method, spontaneous reporting method, literature research method, and medical record review method) in our previous study on post-market clinical safety evaluation of TCMI and other reports, hospital centralized monitoring is a scientific, advanced, and feasible tool to assess clinical safety of TCM injection receiving approval [16]. In this study, monitoring method was improved on the basis of classic hospital-centralized monitoring. First, HIS/LIS system was utilized in the study. To analyze the impact factors of the ADRs/ADEs caused by XSTI, a large number of information associated with ADRs/ADEs were collected. The data collected in the research were composed of general information, medication information and laboratory test data. The general information and medication information such as patients’ ID number, gender, age, height, weight, admission diagnosis, allergic history, nationality, dosage, combined administration were mainly collected by monitoring table, while the laboratory test data such as blood routine, urine routine, faecal routine, liver function, renal function, thrombus and hemostasis and other inspection results were all extracted from HIS/LIS. Besides, the missing information in some of the monitoring tables were also supplemented by the HIS system. Secondly, majority of data collection and analyses in this study was performed by clinical pharmacists. Pharmacist plays important role in rational drug applications and improvement of life quality of patients. In one hand, clinical pharmacists are familiar with the treatment process of ADR/ADEs, on the other hand, they can put more concentration on the research by comparison with clinician. Altogether, pharmacist is the best candidate for ADE surveillance. Last but not least, strict quality control method was designed in this multi-center research. Three-level quality control method used in this study has been successfully used in our previous post-marketing safety surveillance of Danhong injection. In order to strengthen quality control, third-party quality control, a contract research organization (CRO) company, was employed in this study. Because CRO companies has a large number of professional medical and pharmaceutical experts, they actively participate in many phase II or III clinical trials and undertake supervision as the third party, to guarantee the objectivity of the result in studies. Application of above working model in our study contributed greatly to improve objectivity of results and efficiency of research.

Our study has several shorter. We calculated the incidence, main types, main manifestations and severity classification of ADRs in this article, which reflect the safety of clinical use of XSTI in general, but the main influencing factors of ADRs were not studied in this article. However, all relevant data have been collected and are being analyzed. We will complete this part of study in following researches. In addition, the mechanism of the ADRs in the study is still unknown.

## Conclusion

Post-marketing safety surveillance and re-evaluation of XSTI was carried out with 30,884 cases from 33 hospitals in 7 provinces. We obtained incidence rate, types, severities, as well as other information of ADRs/ADEs of XSTI. As far as we know, this research is the first study on the ADR of XSTI using large-scale hospital centralized monitoring method. The results in this study provide a high level evidence-based basis for safety of XSTI. We further founded novel research system and mode of post-marketing safety surveillance and re-evaluation of TCMIs, which also provides a method to dramatically improve rationality and safety of clinical applications of TCMIs.

## Abbreviations

ADE: Adverse Drug Event
ADR: Adverse Drug Reaction
CRO: Contract Research Organization
HIS: Hospital Information System
LIS: Laboratory Information management System
TCMIs: Traditional Chinese medicine injections
XSTI: Xueshuantong injection
